# Synergistic effect of regorafenib with aminoglycosides in ferroptosis-mediated liver injury

**DOI:** 10.3389/fphar.2025.1586578

**Published:** 2025-07-15

**Authors:** Maha Raja Dahar, Shaojun Zhou, Haihong Hu, Jinxiu Lei, Kui Zeng, Lushan Yu

**Affiliations:** ^1^ Institute of Drug Metabolism and Pharmaceutical Analysis, College of Pharmaceutical Sciences, Zhejiang University, Hangzhou, China; ^2^ National Key Laboratory of Advanced Drug Delivery and Release Systems, Zhejiang University, Hangzhou, China; ^3^ Jinhua Institute of Zhejiang University, Jinhua, China

**Keywords:** ferroptosis, regorafenib, antibiotics, ALOX-15, oxidative stress, chemotherapy

## Abstract

**Background:**

Combination therapy of anticancer drugs with antibacterial agents has numerous advantages, but these may develop adverse drug events that were rarely studied. Specifically, regorafenib (REGO) mediates ferroptosis and causes liver injury when used in combination with aminoglycosides (AGs); however, this interaction remains unexplored.

**Methodology:**

The study was conducted using both *in vivo* and *in vitro* assays involving Sprague–Dawley (SD) rats and HepG2/Huh7 to investigate ferroptosis-associated liver injury. The drugs REGO, amikacin (AMK), and gentamicin (GNT) were administered individually as well as in combination to evaluate their noxious effects on the liver. Subsequently, biochemical, histological, and transcriptomic analyses were carried out, and protein expressions were investigated using the immunoblotting assay to explore the mechanisms underlying ferroptotic-mediated liver injury.

**Results:**

The findings of the *in vivo* assay revealed that the combination therapy of regorafenib with aminoglycosides augments alanine transaminase (180%–200%), aspartate aminotransferase (120%–140%), malondialdehyde, and Fe^2+^ levels. It decreases the level of antioxidants and alters histomorphology in SD rats compared to individual therapy. The *in vitro* assay results validate the enhanced levels of cellular iron, lipid peroxidation, reactive oxygen species, and lactate dehydrogenase release, indicating enhanced toxicity. In contrast, it decreased the cell viability ratio, glutathione level, and integrity of the mitochondrial membrane. The real-time quantitative polymerase chain reaction (RT-qPCR) and immunoblotting assay results show downregulation of GPX4 and SLC7A11 expressions, along with elevation of ALOX-15 (3- to 6-fold change).

**Conclusion:**

Thus, combination therapy of regorafenib with amikacin and gentamycin causes significant elevation of iron and lipid peroxidation levels and alteration in protein expressions, which mediates ferroptotic cell death and leads to liver injury.

## Introduction

Drug therapy is remarkably safe and efficacious in many pathophysiological conditions; however, anti-cancer drugs are still associated with numerous adverse drug events (Tewari et al., 2019). Anti-cancer drugs are multimodal approaches in oncological treatment, involving highly complex regimens that result in a high susceptibility toward their noxious effects (Chopra et al., 2016). Chemotherapeutic drugs are frequently linked to chemotherapy-induced myelosuppression, which leads to severe hematological complications (Weiss, 2012) like thrombocytopenia, leukemia, and persistent anemia, specifically long-term neutropenia, and immunosuppression (Vial and Descotes, 2003). These complications enhance the risk of infections even during or after chemotherapy (Ba et al., 2020; Blayney and Schwartzberg, 2022; Dessalegn et al., 2023).

Regorafenib (REGO), a vascular endothelial growth factor receptor tyrosine kinase inhibitor, involves the activity of several kinase proteins and is associated with the tumor microenvironment, oncogenesis, and angiogenesis (Wilhelm et al., 2011). REGO was approved for treatment of metastatic colorectal cancer, advanced hepatocellular carcinoma (Granito et al., 2021), breast cancer (Salahuddin et al., 2021), esophageal cancer (Cytryn et al., 2023), etc. In 2,341 subjects, REGO-associated hematological toxicities include 22% thrombocytopenia, 3% anemia, and 10% neutropenia. In this scenario, the use of REGO dramatically elevated the risk of hematological toxicities, causing a higher risk of developing severe infections (Zhao and Zhao, 2017). Moreover, REGO influences the tumor microenvironment by modulating the activity of various immune cells, which can contribute to an overall immunosuppressive environment (Ou et al., 2021; Liu et al., 2022). It was proven that the drugs affecting the vascular endothelial growth factor pathway could alter immune cell function; for instance, REGO may inhibit the function of natural killer cells and T lymphocytes, which are critical for reducing sepsis. In clinical settings, patients receiving REGO have been reported to experience higher rates of serious infections (Kim et al., 2024). Numerous studies have reported that an unknown infection is suspected during or after cytotoxic chemotherapy, so certain broad-spectrum antibiotics are recommended (Moon et al., 2006; Taplitz et al., 2018; Jayanth et al., 2021).

Aminoglycosides (AGs) are broad-spectrum bactericidal antibiotics primarily used to treat various infections caused by aerobic and anaerobic organisms. They are commonly used in chemotherapy settings and are highly recommended, particularly when patients have a low neutrophil count (Taplitz et al., 2018). Among all AGs, gentamicin (GNT) or amikacin (AMK) is used concomitantly with various chemotherapeutic drugs (Zumla, 2010; Avent et al., 2011), like vinblastine (Cuccarese et al., 2013), cisplatin (Schacht et al., 2012), and methotrexate (Mendu et al., 2007), to enhance the efficacy and support defense mechanisms from certain types of infections. The use of antibiotics with cytotoxic chemotherapeutic drugs may be beneficial in terms of defense mechanisms; however, despite the potential for adverse drug events, this combination has been rarely studied. Specifically, AGs used with REGO mediate ferroptosis and cause liver injury; however, this interaction remains unexplored.

In recent years, the research on ferroptosis has grown exponentially. This type of regulated cell death is primarily induced by iron-dependent phospholipid peroxidation, which is controlled by a number of metabolic processes intracellularly, via System Xc^−^, the mevalonate pathway, Nrf2, p53, and other pathways (Ou et al., 2022). Ferroptosis is distinct from necrosis, autophagy, apoptosis, and other types of controlled cell death in terms of the genetics, biochemistry, and appearance (Dixon et al., 2012). As a major kind of cell death, ferroptosis has received increased attention in both clinical and basic research works. Ferroptosis, which involves a number of substances, both as triggers and inhibitors, severely impacts the physiological system, including the respiratory system (Yu and Sun, 2023), renal system (Zhou et al., 2022), gastrointestinal system, and hepatic system. Furthermore, the effect of ferroptosis on several kinds of diseases have also been extensively studied; for instance, cardiovascular illnesses (Guo et al., 2022; Cai et al., 2023), neurodegenerative problems (Ren et al., 2020), liver illnesses (Wu et al., 2021), and bone-associated diseases (Chen et al., 2023) may either be acute or chronic (Chen F et al., 2024). The proposed research work includes *in vivo* and *in vitro* biological assays. It aims to investigate the mechanism underlying ferroptosis-associated liver injury mediated by the combination therapy of anticancer drugs, specifically following the use of regorafenib with amikacin (R + A) and regorafenib with gentamicin (R + G).

## Materials and methods

### Chemicals and reagents

STIVARGA tablets (regorafenib) were obtained from Bayer Pharmaceuticals Ltd., Leverkusen, Germany. Amikacin sulfate injection was purchased from Zhejiang Cheng Yi Pharmaceutical Co. Ltd., Shanghai, China. Gentamicin sulfate injection was obtained from Reyoung Pharmaceuticals, Shandong, China. Alanine transaminase (ALT), aspartate aminotransferase (AST), lipid peroxidation (LP), and ionic iron assay kits were provided by Nanjing Jiancheng Bioengineering Institute, China. Malondialdehyde (MDA), glutathione (GSH), superoxide dismutase (SOD), catalase (CAT), 2-7 dichlorodihydrofluorescein diacetate (DCFH-DA), reactive oxygen species (ROS), lactate dehydrogenase (LDH), and JC-1 assay kits were obtained from Beyotime Biotechnology Co., Ltd., Shanghai, China. Ferrostatin-1 was purchased from MedChemExpress, United States. The cell count kit-8 (CCK-8) assay kit (Cat # CK001) was provided by LAB LEAD, China. The primary antibodies including Alox-15 (Cat # SC133085) were obtained from Santa Cruz biotechnology, TX, United States, and p53 (Cat # 60283-2lg), Stat-1 (Cat # 10708-1-AP), Gpx4 (Cat # 67763-1-lg), Slc7a11 (Cat # 26864-1-AP), GAPDH (Cat # 60004-1-IG), and secondary antibodies were obtained from Proteintech, China.

### Animal strains and care

Sprague–Dawley (SD) male rats, weighing 200–225 g and aged 6–8 weeks, were purchased from Hangzhou Medical College, Zhejiang, China “Zhejiang Academy of Medical Sciences” (SCXK–Zhejiang 2019-0002). The animals were housed in plastic cages and had free access to nutrition and were exposed to equal light and dark cycles during the day to facilitate the experimental procedures. Animal handling ethics protocols were followed as per approved institutional guidelines established by the Zhejiang University Institutional Animal Care. The animals were kept under a controlled temperature of 20–25°C ± 2°C and 40%–60% humidity. After acclimatization and feeding for 7 days, the rats were randomly distributed into six groups, each with six animals, and treated with individual and combination therapies of REGO, AMK, and GNT as follows:

• Co: negative control group (1.5 mL of CMC administered orally and 0.5 mL 0.9% sodium chloride administered intraperitoneally).
• REGO: regorafenib alone at 15 mg kg^−1^ once daily.
• AMK: amikacin injection alone at 15 mg kg^−1^ once daily.
• GNT: gentamicin injection alone at 15 mg kg^−1^ once daily.
• R + A: regorafenib: 15 mg kg^−1^ and amikacin: 15 mg kg^−1^ once daily.
• R + G: regorafenib: 15 mg kg^−1^ and gentamicin: 15 mg kg^−1^ once daily.

REGO was administered via oral gavage for 21 days. AMK/GNT treatments began on the 19th day and were administered intraperitoneally (IP). After the completion of the whole trial, animals were sedated with ketamine hydrochloride injection (45 mg kg^−1^) and xylene (5 mg kg^−1^) and then sacrificed by cervical dislocation. Blood samples were drawn from the inferior vena cava. The serum/plasma from whole blood was collected through centrifugation at 5,000 × *g* at 4°C for 10 minutes and kept at −20°C for further biochemical analysis. The liver was dissected and longitudinally bisected; one-half of the liver tissues from different groups were preserved in 4% paraformaldehyde for histomorphological analysis, and the remaining portions of the liver were frozen at −80°C for further experimental purposes.

### Cell culture

An *in vitro* assay was performed to analyze the further mechanism of liver injury mediated by the combination therapy of REGO with AMK and GNT. For this purpose, HepG2 and Huh7 were obtained from the Chinese Academy of Sciences, Shanghai, China. The cells were cultured in Dulbecco’s modified Eagle medium (DMEM), supplemented with fetal bovine serum (FBS) 10% (v/v), and incubated at 37°C in an incubator containing 5% CO_2_. Subsequently, the respective cells were exposed to individual and combination therapies and grouped as follows:

• Co: DMSO (0.01%) and considered the negative control.
• REGO: regorafenib 2 µM.
• AMK: amikacin 10 µM.
• AMK: amikacin 20 µM.
• GNT: gentamicin 10 µM.
• GNT: gentamicin 20 µM.
• R + A: regorafenib: 2 μM + amikacin: 10 μM.
• R + A: regorafenib: 2 μM + amikacin: 20 μM.
• R + A + F: regorafenib: 2 μM + amikacin: 20 μM + ferrostatin-1: 10 μM.
• R + G: regorafenib: 2 μM + gentamycin: 10 μM.
• R + G: regorafenib: 2 μM + gentamycin: 20 μM.
• R + G + F: regorafenib: 2 μM + gentamicin: 20 μM + ferrostatin-1: 10 μM.

### Analysis of liver function biomarkers, oxidative stress determinants, and iron contents

To determine the levels of ALT, AST, MDA, GSH, SOD, and CAT, the liver tissue, the blood of SD rats, HepG2 cells, and Huh7 cells were homogenized. Subsequently, these were centrifuged at 10,000–12,000 x g for 15 min at 4°C, and a supernatant solution was collected. Further protocols were followed as per the manufacturer’s instructions provided with the commercial assay kits.

### Histopathological analysis of the liver injury

After 24 h of fixation in 4% paraformaldehyde, the hepatic tissues of SD rats were embedded in paraffin. Subsequently, the tissues were sliced into 4-mm pieces and stained with hematoxylin and eosin (H and E). Finally, each section was examined under the optical microscope. According to liver H and E staining, Suzuki’s criteria (Suzuki et al., 1993) were followed to determine the severity of liver damage.

### Immunoblotting analysis

A “One Step PAGE Gel Fast Preparation Kit” (Vazyme, Cat #E303-C1) was used to prepare polyacrylamide gels and electrophoretic separation of proteins. Primary antibodies, including Slc7a11, Gpx4, p53, Stat-1, Alox-15, and Gapdh, were used after preparing the primary antibody’s dilution buffer provided by Beyotime Biotechnology, People’s Republic of China. The specific dilution ratio of various antibodies is presented in Supplementary Table S1.1. Finally, images were taken using the Azure Bioanalytical Imaging System (Azure 300, United States) with system-provided software. The scanned digital images were quantified using NIH ImageJ software.

#### Cellular viability and lactate dehydrogenase release analyses

Cell viability was quantified using cell count kit-8 (CCK-8) provided by LAB LEAD, Beijing, China (Cat # CK001), and LDH release toxicity was investigated using an assay kit supplied by Beyotime, China (Cat #C0016). HepG2 and Huh7 cells were incubated at a density of 1.0 × 10^6^ in 96-well plates. Subsequently, cells were exposed to the individual as well as combination treatment of the corresponding drugs for the next 24 h. Further procedures were carried out according to the manufacturer’s instructions. Finally, the cell viability was measured at 450 nm and LDH activity at 490 nm using a Thermo Scientific NanoDrop 2000 UV–Vis spectrophotometer.

### Reactive oxygen species analysis

To identify the cellular ROS level in HepG2 cells, a DCFH-DA assay kit was used, which was provided by Beyotime, China (Cat #S0033s). The cells were exposed to corresponding drugs for 24 h. Subsequent steps were followed according to the manufacturer’s instructions. The final results were analyzed using a fluorescence microscope Nikon Eclipse Ti (Tokyo, Japan).

### Real-time quantitative polymerase chain reaction assay

TRIzol reagent obtained from TIANGEN Biotech Co., Ltd., Beijing, China (Cat # DP424) was used to obtain the total RNA from HepG2 cells, Huh7 cells, and liver tissues. The RNA was then reverse-transcribed onto cDNA using a commercial kit. Real-time quantitative polymerase chain reaction (RT-qPCR) was performed using SYBR Green (Rosh, United States) on an Applied Biosystems system by Thermo Fisher Scientific, United States. The final results were evaluated using the 2^(-△△CT) method. GAPDH is considered a control gene. The reaction system established for RT-qPCR and specific primers for each gene are enlisted in Supplementary Table S1.2, 1.3A, 1.3B.

#### Mitochondrial membrane potential determination

The mitochondrial membrane potential (MMP) was determined following instructions for the JC-1 dye assay kit purchased from Beyotime, China (Cat #C2006). In short, HepG2 cells were incubated in equal density in a 6-well plate and exposed to drugs for 24 h. Subsequently, the respective treated cells of every group were rinsed with PBS and incubated with 1,000 μL of the JC-1 working solution for 20 min at 37°C. Finally, the cells were analyzed using a Nikon A1R confocal microscope (Tokyo, Japan).

### Statistical analysis

The experiment's numerical data was expressed as ± SD, further analysis was performed using GraphPad Prism 8.1.2. The values with (*) are significantly different from the Co (negative control) using ANOVA and Dunnet's technique (*p < 0.05), (**p < 0.005), (***p < 0.001), (****p < 0.0001) and ns means non-significant.

## Results

### The combination therapy of REGO with AGs induces liver injury in SD rats

**FIGURE 1:**
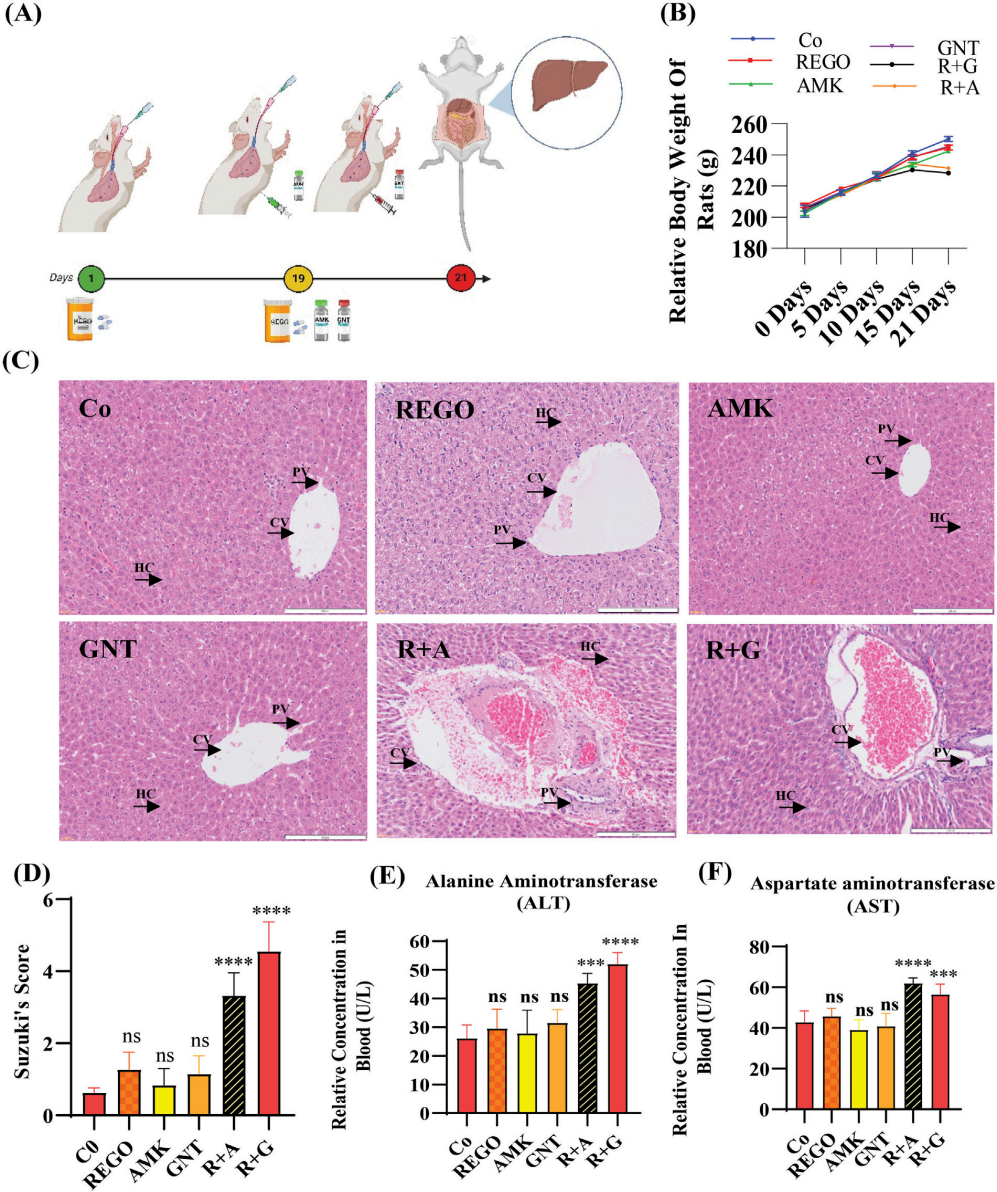
The combination therapy of REGO with AMK and GNT enhanced liver injury in SD rats. After completing 21 days of treatment, blood and liver tissues were analyzed. **(A)** Study protocol for the animal-based study; **(B)** physical body weight analysis; **(C)** images of H and E staining of the liver. **(D)** H and E score according to Suzuki’s study. **(E)** ALT, **(F)** AST in each group. Data are represented as mean ± SD (n = 6). * symbolizes a significant variation relative to the Co. The value represents *p < 0.05, while **p < 0.005, ***p < 0.001, and ****p < 0.0001 are considered significant, and ns means non-significant.

To identify the hepatotoxic effects of combination therapy of REGO with AGs, specifically AMK and GNT, the drugs were administered to SD rats. The findings revealed that the body weight was decreased in SD rats when combination therapy was started, specifically in R + A and R + G groups of rats (Figure 1B). However, other groups that were treated with monotherapy having the same body weight were the negative control group. The microscopic images of H and E staining revealed that the drug monotherapy has no impact on the liver. It is noteworthy that severe alterations were observed at the site of hepatic cells (HCs) in groups treated with combination therapy (R + A and R + G groups), characterized by inflammation, cytoplasmic vacuolation, and microvascular injury at the central vein (CV) and portal vein (PV). It disrupted the cellular architecture and histological architecture, as shown in Figure 1C. Meanwhile, the level of ALT was enhanced in R + A significantly (p < 0.001) and also in R + G (p < 0.0001), while the AST level increased in R + A (p < 0.0001) and in R + G (p < 0.001) compared to the Co group. (Figures 1E,F). In contrast, no changes were observed in groups treated with individual therapies of REGO, AMK, and GNT. These findings indicate that combination therapy of REGO with AMK and GNT induces liver injury in SD rats.

### The combination therapy of REGO with AGs induces oxidative stress and mediates ferroptosis in SD rats

**FIGURE 2:**
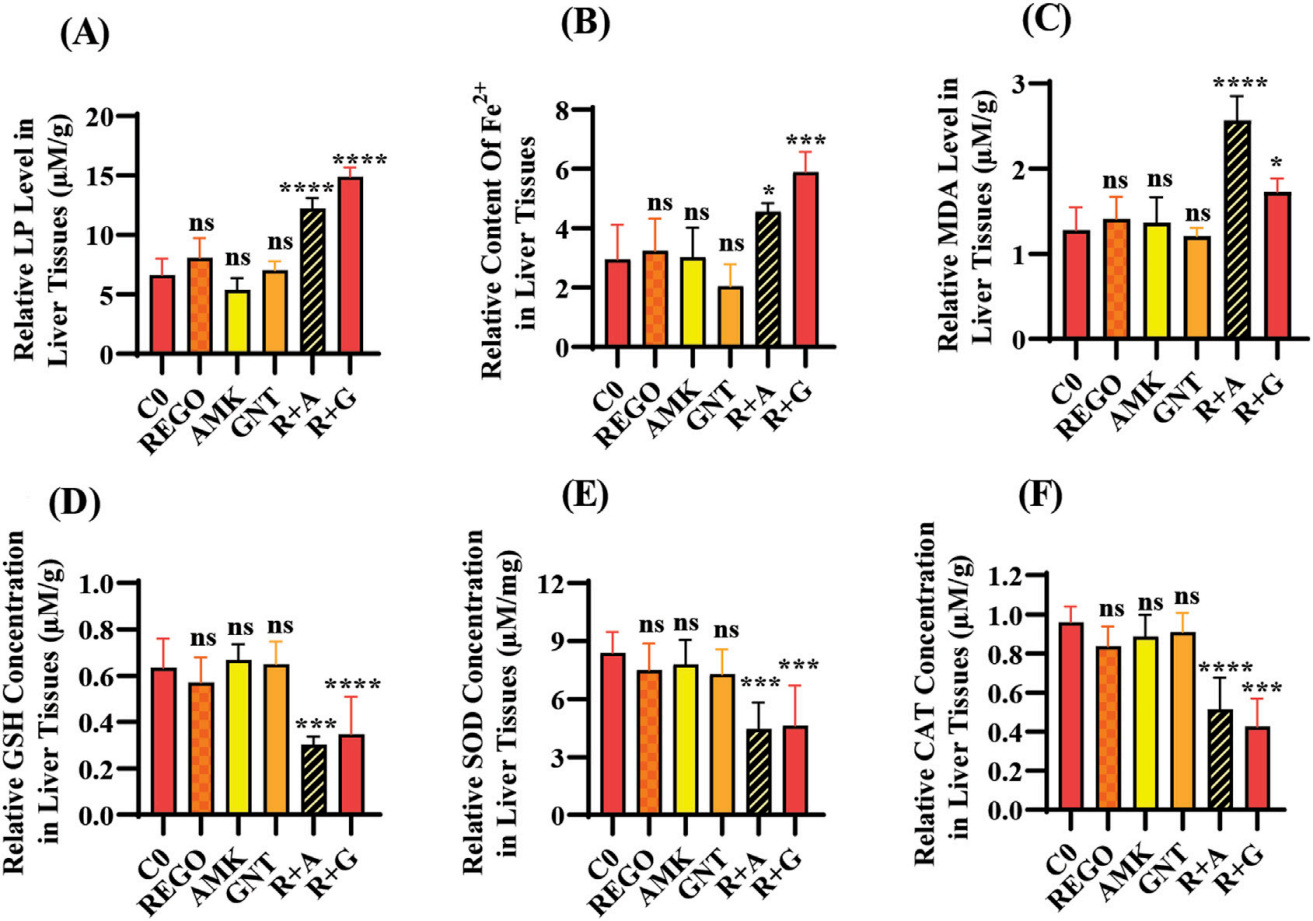
The combination therapy of AMK and GNT with REGO induces oxidative stress through elevating LP, ionic iron, and MDA levels in liver tissues of SD rats after completion of 21-day treatment. Subsequently, these parameters were determined on liver tissues of SD rats: **(A)** lipid peroxidation (LP); **(B)** level of ionic iron; **(C)** MDA level; **(D)** GSH; **(E)** superoxide (SOD); **(F)** catalase (CAT) level. The data are represented as mean ± SD (n = 3). * symbolizes a significant variation relative to the Co. The value represents *p < 0.05, while **p < 0.005, ***p < 0.001, and ****p < 0.0001 are considered significant, and ns means non-significant.

**FIGURE 3:**
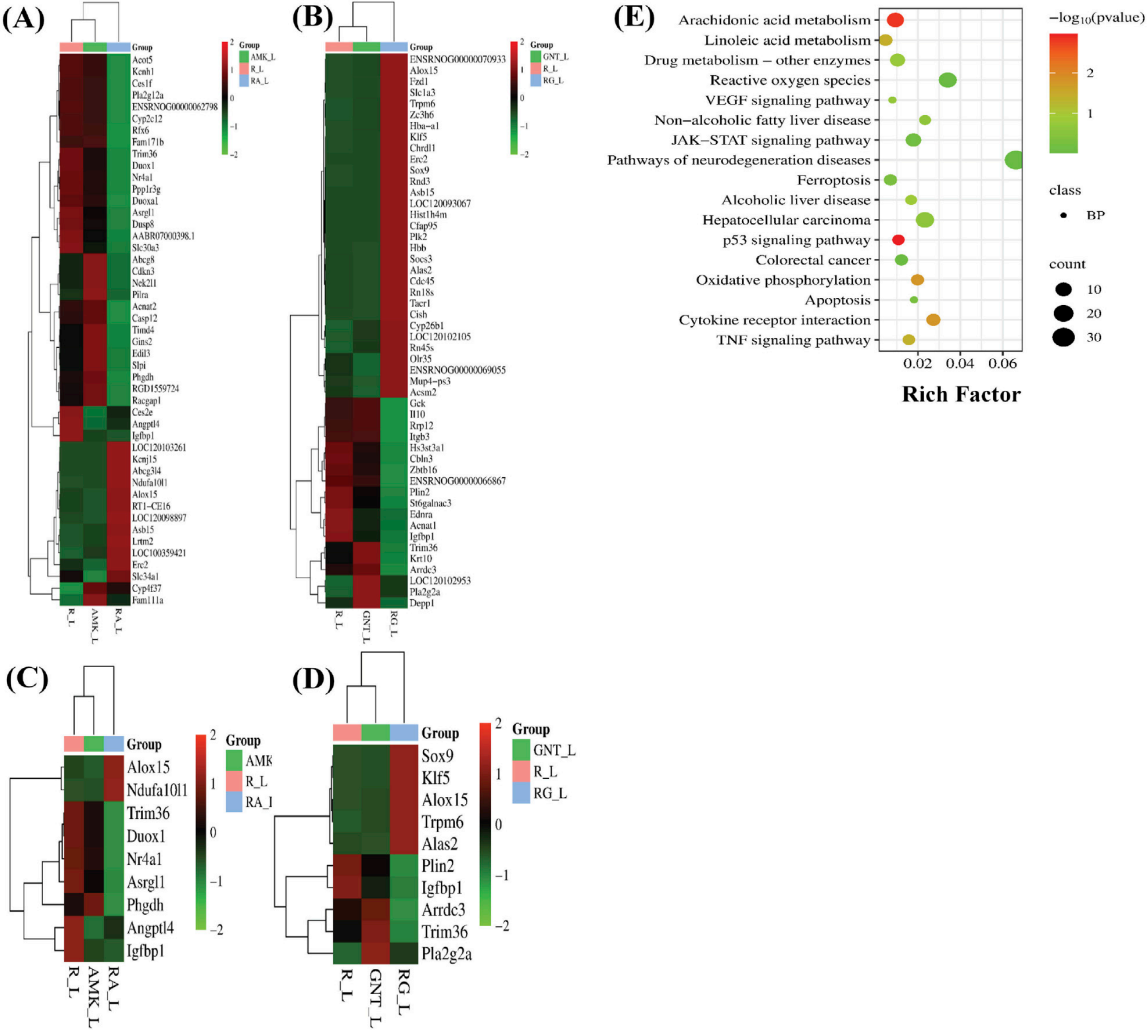
Following individual and combined use of corresponding drugs, liver tissues of SD rats were analyzed through RNA-seq. The data show the following: **(A)** heat map of DEGs in REGO vs. AMK groups; **(B)** heat map of DEGs in REGO vs. GNT groups; **(C)** heat map of DEGs associated with ferroptosis in REGO vs. AMK groups; **(D)** heat map of DEGs associated with ferroptosis in REGO vs. GNT groups; **(E)** KEGG enrichment factor showing 20 common pathways involved in combination therapy of REGO vs. AMK and REGO vs. GNT groups. R, regorafenib alone; AMK, amikacin alone; GNT, gentamicin alone; RA, combination therapy of REGO with amikacin; RG, regorafenib with gentamicin; L, liver tissues.

**FIGURE 4:**
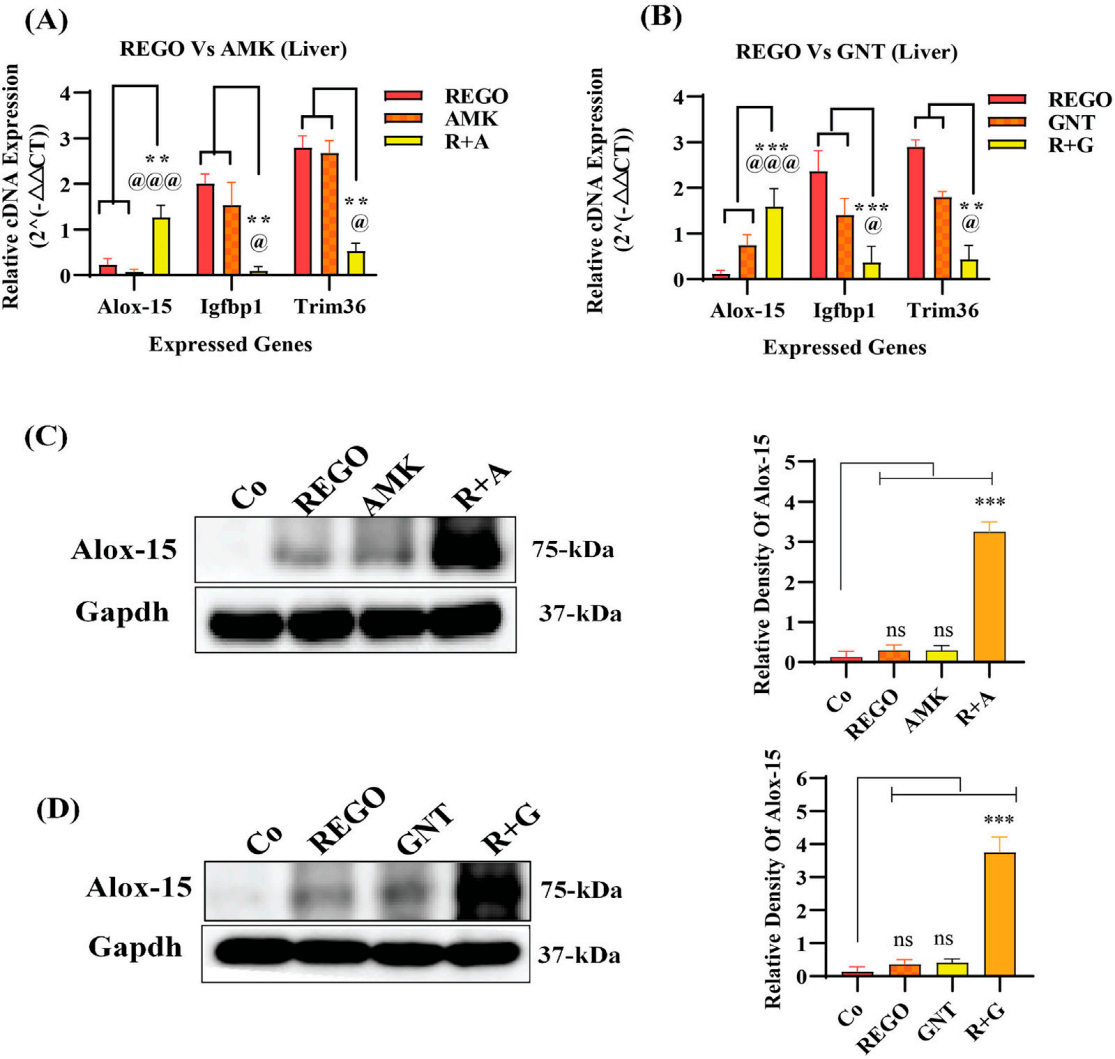
Validation of RNA-seq analysis through RT-qPCR and immunoblot. **(A)** cDNA expression of Alox-15 in REGO vs. AMK groups; **(B)**- cDNA expression of Alox-15 in REGO vs. GNT groups; **(C)** protein expression of Alox-15 in REGO vs. AMK groups; **(D)**- protein expression of Alox-15 in REGO vs. GNT groups. The data are represented as mean ± SD. * symbolizes a significant variation relative to the Co, and @ value represents a significant difference relative to AMK or GNT. The value represents @,*p < 0.05, while @@, **p < 0.005, @@@, ***p < 0.001, and @@@@, ****p < 0.0001 are considered significant, and ns means non-significant.

Increased levels of LP, ionic iron, and oxidative stress are considered biochemical hallmarks of ferroptosis (Chen et al., 2021). To investigate the mechanism underlying liver injury caused by the combination therapy of REGO with AGs, tissues of SD rats were used following individual and combination treatments with the corresponding drugs. The findings revealed that the LP level was significantly increased in R + A and R + G groups (p < 0.0001), while the level of ionic iron was also elevated in the R + A (p < 0.05) and R + G (p < 0.001) groups. The MDA level was significantly increased in the R + A (p < 0.0001) and in R + G (p < 0.05) groups. In contrast, the concentrations of GSH, CAT, and SOD were significantly reduced in the R + A and R + G groups. However, no significant difference was observed in REGO, AMK, and GNT groups that received individual therapy for the corresponding drugs (Figures 2A–F). Furthermore, transcriptomic analysis was performed as follows: raw counts were normalized using DESeq2’s median-of-ratios method to compute sample-specific size factors, and differential gene expression analysis was performed using Wald’s test, and p-values were adjusted for multiple testing using the Benjamin–Hochberg FDR method with a cut-off of FDR <0.05 │log2FC│. The findings revealed that 887 genes were dysregulated, of which 454 were upregulated and 433 were downregulated. Subsequently, 50 genes were selected from each group, and those were most differently expressed genes (DEGs) between the treatment groups (Figures 3A,B). DEGs are involved in ferroptosis in the respective groups, including *Alox-15* (arachidonate 15 lipoxygenase), *Ndufa10l1* (NADH dehydrogenase-ubiquinone-1 alpha sub-complex 10-like-1), *Angptl4* (angiopoietin-like 4), *Nr4a1* (nuclear receptor subfamily 4 group A-1), *Asrgl1* (asparaginase and isoaspartyl peptidase-1), *Trim36* (tripartite motif-containing-36), *Phgdh* (phosphoglycerate dehydrogenase), *Duox1* (dual oxidase-1), *Igfbp1*(insulin-like growth factor-binding protein-1) (Figure 3C), *Alox-15* (arachidonate 15 lipoxygenase), *Sox9* (SRY-Box transcription factor-9), *Trpm6* (transient receptor potential cation channel subfamily-M-6), *Klf5* (Kruppel-like factor-5), *Alas2* (5′-aminolevulinate synthase 2), *Pla2g2a* (phospholipase A2, group IIA), *Arrdc3(*arrestin domain-containing 3), *Plin2* (perilipin 2), *Trim36* (tripartite motif-containing-36), and *Igfbp1*(insulin-like growth factor-binding protein-1) (Figure 3D). The Kyoto Encyclopedia of Genes and Genomes (KEGG) pathway-rich factor shows that ferroptosis pathways are involved in the top 20 pathways (Figure 3E). From these investigations, it was summarized that after the combination treatment of REGO with AGs, *Alox-15* was found to be commonly expressed in all groups and significantly upregulated, which is associated with the ferroptosis pathway. To validate the expression profiling of *Alox-15* and other DEGs, RT-qPCR was performed (Figures 4A,B). Additionally, the protein expression of *Alox-15* was analyzed through an immunoblot assay (Figures 4C,D). Consistent with the results of RNA-seq, RT-qPCR, and immunoblotting assay, it was confirmed that *Alox-15* was significantly overexpressed when REGO was concomitantly used with AMK and GNT, which may induce ferroptosis.

### The combination therapy of REGO with AGs reduced cell viability, mitochondrial membrane potential, and increased LDH release in HepG2 and Huh7 cells

**FIGURE 5:**
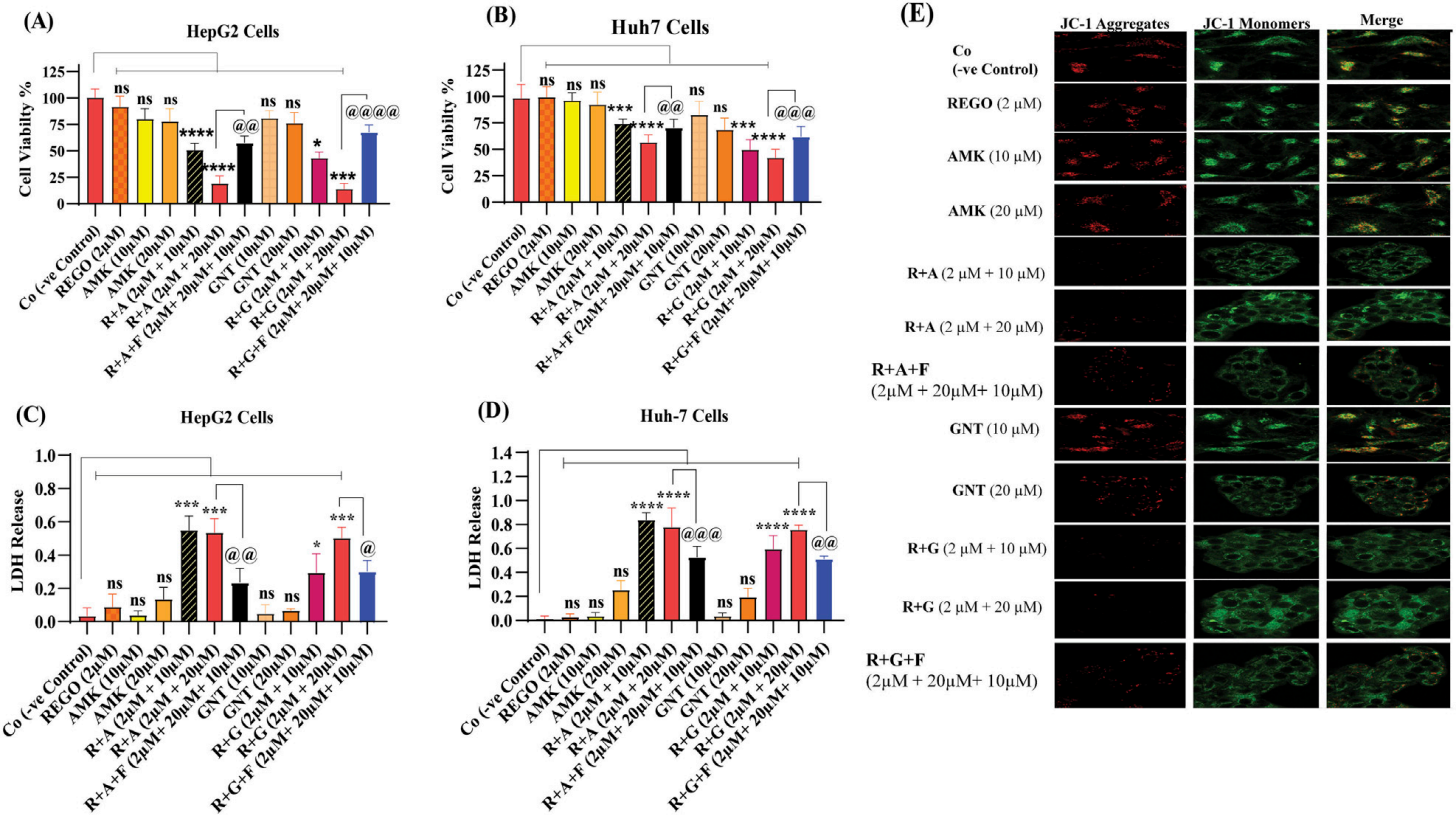
REGO and its combination treatment with AMK and GNT decreases the cell viability and MMP levels and increases LDH release in Huh7 and HepG2 cells. **(A)** The ratio of cell viability in HepG2 cells. **(B)** The ratio of cell viability in Huh7 cells. **(C)** LDH release in HepG2 cells. **(D)** LDH release in Huh7 cells. The data are represented as mean ± SD (n = 3); * symbolizes a significant variation relative to the Co, and ^@^ indicates significant variation as compared to R + A and R + G accordingly. The value represents ^@^, *p < 0.05, while @@, **p < 0.005, @@@, ***p < 0.001, and @@@@, ****p < 0.0001 are considered significant, and ns means non-significant. **(E)** JC-1 aggregates. Red represents the healthy mitochondria, and green represents damaged mitochondria. The red/green fluorescence ratio provides a sensitive measure of mitochondrial function and integrity relevant to the ferroptotic process. The images were captured using a ×60 scale bar.

The aforementioned outcomes of the *in vivo* assay encourage us to investigate further mechanisms of combination therapy of REGO with AGs, which may be involved in hepatic injury. For this purpose, an *in vitro* assay using HepG2 and Huh7 cells was established. Through this, it is determined that the combination therapy of corresponding drugs (R + A and R + G) reduces the cell viability ratio according to the concentration gradient of drugs. Meanwhile, individual therapy showed a slight change in the cell viability, but this was insignificant as compared to Co (Figures 5A,B). Accompanied with the decreased ratios of cell viability, an increased LDH release was observed in R + A and R + G groups of HepG2 and Huh7 cells with exposure to different drug concentrations (Figures 5C,D). To examine MMP levels in response to the combination therapy of REGO with AGs, JC-1 staining was used. It is noteworthy that the MMP level remarkably decreased after combination therapy of corresponding drugs, i.e., in R + A and R + G groups of cells compared to Co, while cells exposed to individual therapy, i.e., REGO, AMK, and GNT, have no changes (Figure 5E). Additionally, in the presence of fer-1 with combination therapy of REGO with AMK (R + A + F) and REGO with GNT (R + G + F), cell viability and MMP were improved, and LDH release was decreased. These key outcomes validate that the combination therapy of REGO with AGs mediates ferroptosis, which leads to liver injury.

### The combination therapy of REGO with AGs mediated ferroptosis in HepG2 and Huh7 cells

**FIGURE 6:**
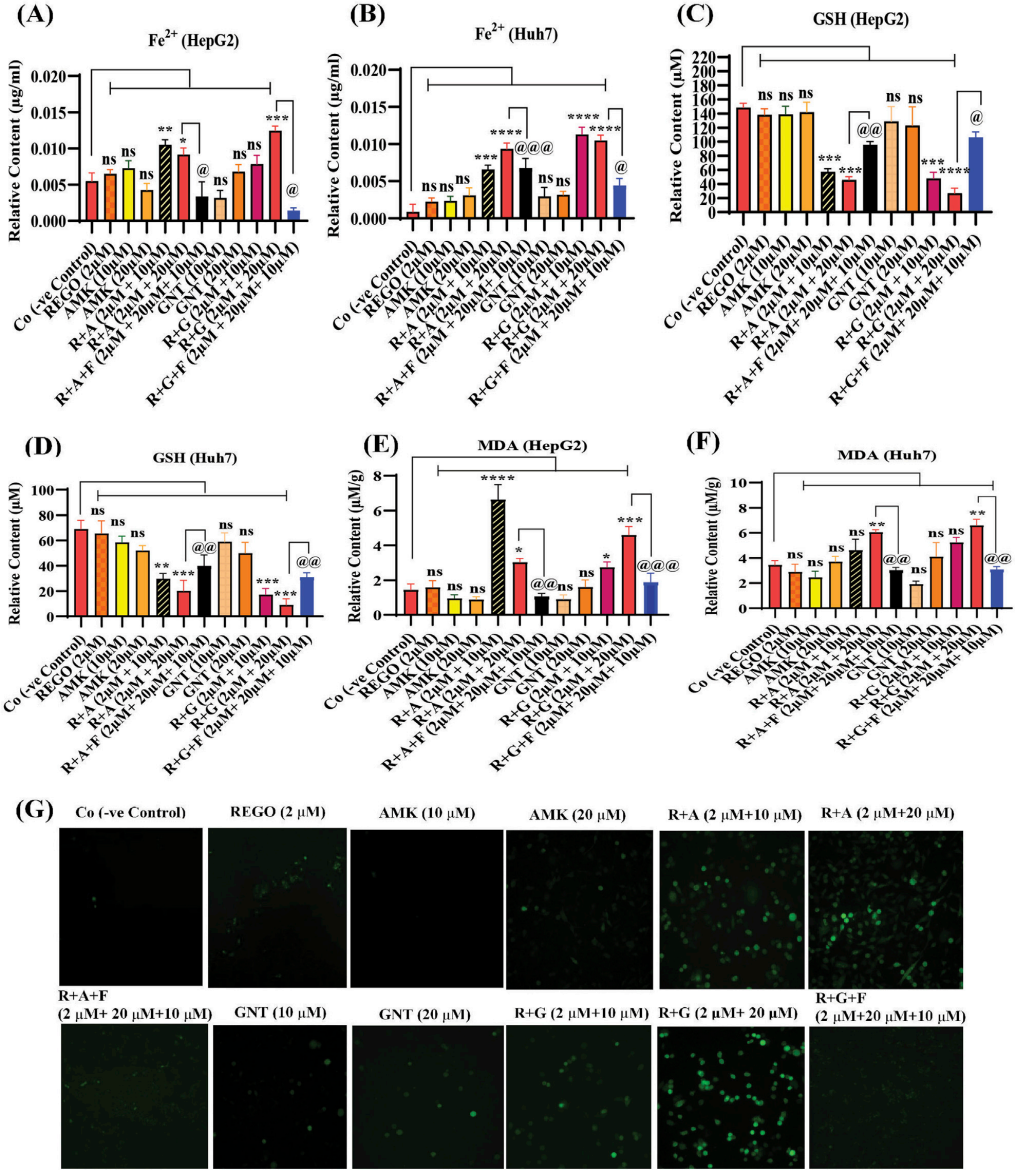
REGO mediates ferroptosis in HepG2 and Huh7 cells, when it was used with AMK and GNT. The cells were exposed to respective drugs for 24 h; meanwhile, it increases the levels of ionic iron, MDA, and ROS and decreases the GSH level. **(A)** Ionic level of iron in HepG2 cells; **(B)** ionic level of iron in Huh7 cells; **(C)** GSH in HepG2 cells; **(D)** GSH in Huh7 cells; **(E)** MDA in HepG2 cells; **(F)** MDA in Huh7 cells. The data are represented as mean ± SD (n = 3). * symbolizes a significant variation relative to the Co, and ^@^ indicates significant variation compared to R + A and R + G groups accordingly. The value represents @, *p < 0.05, while @@, **p < 0.005, @@@, ***p < 0.001, and @@@@, ****p < 0.0001 are considered significant, and ns means non-significant. **(G)** Higher green fluorescence depicts increased ROS levels (the images were captured using ×20 scale bar).

**FIGURE 7:**
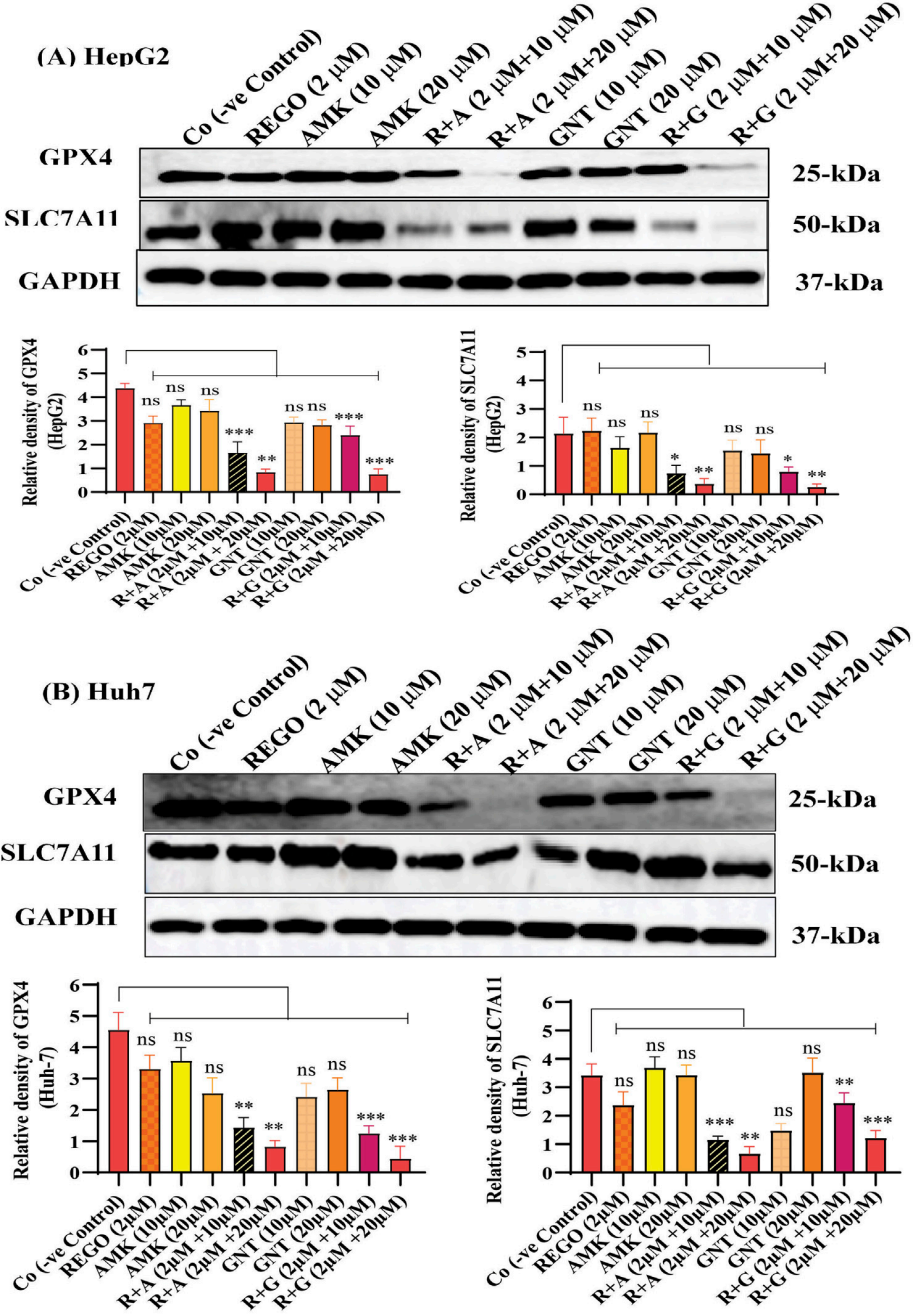
REGO downregulates protein expressionS of GPX4 and SLC7A11, when it was combined with AMK and GNT. The liver cells were exposed to respective drugs for 24 h; meanwhile, they were analyzed through immunoblotting assay. **(A)** Protein expressions of GPX4 and SLC7A11 in HepG2 cells. **(B)** Protein expressions of GPX4 and SLC7A11 in Huh7 cells. * shows a significant variation relative to the Co. The data are represented as mean ± SD. * symbolizes a significant variation relative to the Co; the value represents *p < 0.05, while **p < 0.005, ***p < 0.001, and ****p < 0.0001 are considered significant, and ns means non-significant.

**FIGURE 8:**
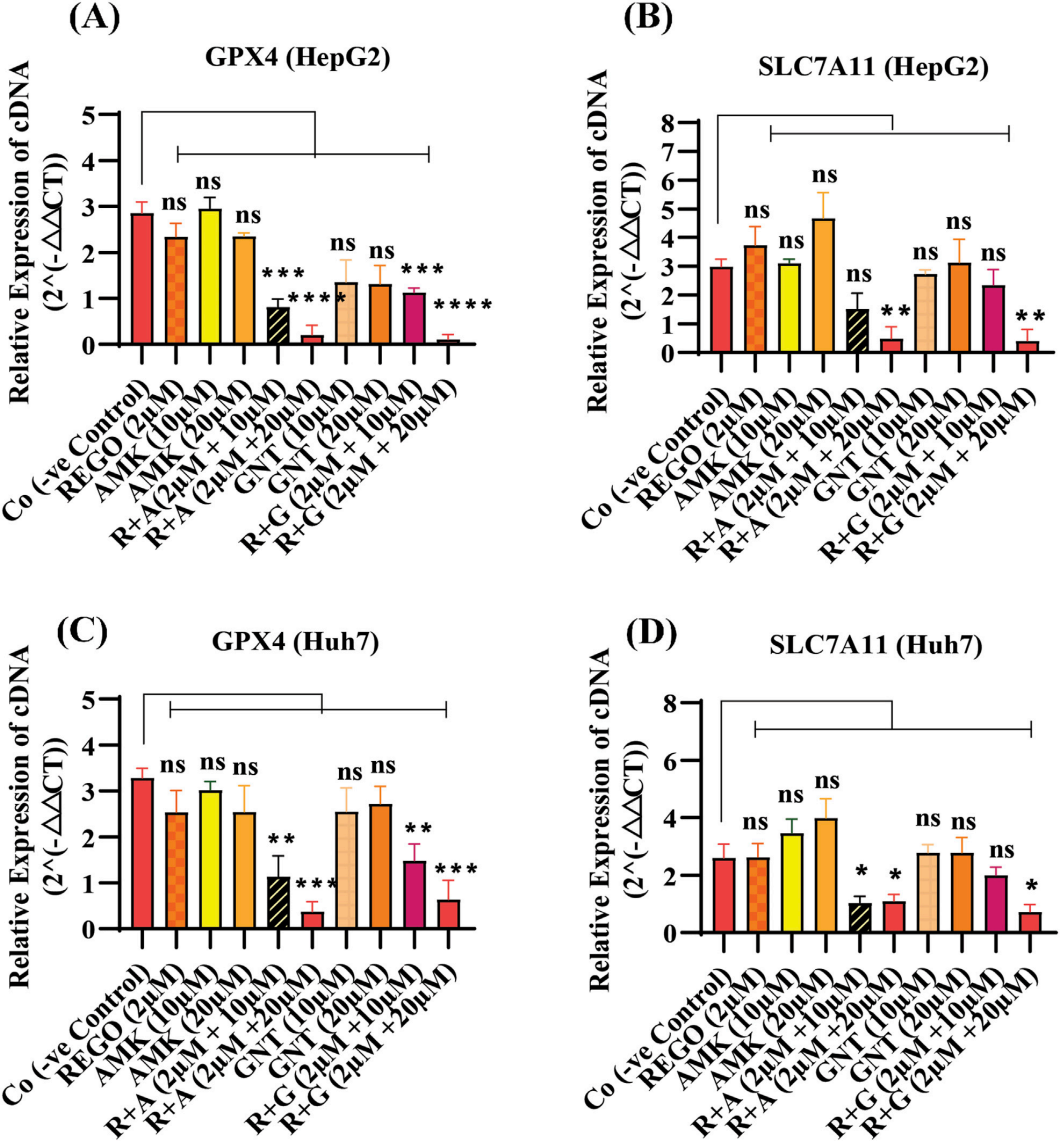
REGO downregulates gene expressions of GPX4 and SLC7A11 in HepG2 and Huh7 cells, when it was combined with AMK and GNT. The cells were exposed to respective drugs for 24 h; subsequently, the cDNA level was investigated through RT-qPCR. **(A)** Fold change of GPX4 in HepG2 cells; **(B)** fold change of SLC7A11 in HepG2 cells; **(C)** fold change of GPX4 in Huh7 cells; **(D)** fold change of SLC7A11 in Huh7 cells. The data are represented as mean ± SD (n = 3). * symbolizes a significant variation relative to the Co. The value represents *p < 0.05, while **p < 0.005, ***p < 0.001, and ****p < 0.0001 are considered significant, and ns means non-significant.

Iron accumulation and elevated LP levels are considered phenotypic features, while downregulation of GPX4 and SLC7A11 expressions are considered genotypic hallmarks of ferroptosis (Capelletti et al., 2020). Notably, it was extensively studied that iron accumulation increases ROS levels, thereby elevating oxidative stress and potentiating liver fibrosis (Mehta et al., 2019). Consistently, iron levels in REGO-, AMK-, and GNT-treated HepG2 and Huh7 cells were examined. Meanwhile, it was observed that the combination therapy of REGO with AMK and GNT augments iron accumulation intracellularly. Specifically, Fe^2+^ was significantly elevated in HepG2 cells (p < 0.005) in R + A (2 μM + 10 μM) and in R + G (2 μM + 20 μM) groups (p < 0.001), as depicted in Figure 6A. Meanwhile, in Huh7 cells, it was enhanced significantly (p < 0.0001) in R + A (2 μM + 20 μM) and R + G (2 μM + 20 μM) groups, as displayed in Figure 6B. MDA, GSH, and ROS levels in hepatocytes were also analyzed; the findings show elevated ROS and MDA levels in R + A and R + G groups. However, GSH levels markedly reduced in HepG2 and Huh7 cells in R + A and R + G groups at both extents, as compared to the negative control group. In contrast, slight changes were observed in cell groups that were exposed to AMK, REGO, and GNT alone, but these changes were not significant (Figures 6C–G). Notably, in the presence of fer-1, the levels of Fe^2+^, MDA, and ROS were significantly decreased in HepG2 and Huh7 cells, while the GSH level was significantly improved. Consistently, it was also determined that REGO and its combination therapy extensively downregulates the proteins levels of GPX4 and SLC7A11 in HepG2 and in Huh7 cells (Figures 7A,B). Meanwhile, the results of RT-qPCR show that the fold change of GPX4 and SLC7A11 was significantly decreased in HepG2 cells, specifically in R + A and R + G groups at various extents (Figures 8A,B). Simultaneously, in Huh7 cells, the expression level of GPX4 was also reduced in R + A and R + G groups, as displayed in Figure 8C, while SLC7A11 level is significantly decreased in both R + A and R + G groups as the drug content was increased (Figure 8D). The overall findings demonstrate that when REGO is co-administered with AMK and GNT, it induces ferroptosis, which may lead to liver injury.

### The combination therapy of REGO with AGs activates ALOX-15, which is involved in hepatic ferroptosis

**FIGURE 9:**
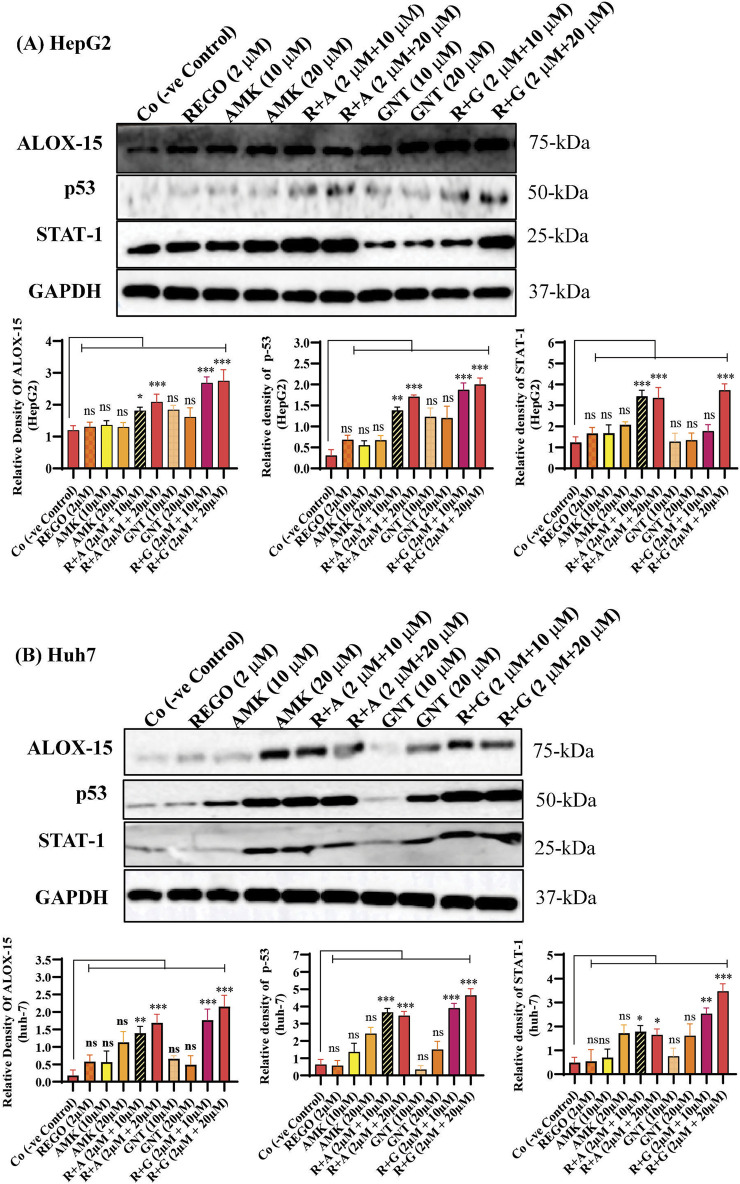
The combination therapy of REGO with AMK and GNT elevates ALOX-15 expression. Cells were exposed to respective treatments for 24 h **(A)** Protein expression of ALOX-15, p53, and STAT-1 in HepG2 cells. **(B)** Protein expressions of ALOX-15, p53, and STAT-1 in Huh7 cells, investigated through immunoblot assay. The data are represented as mean ± SD. * symbolizes a significant variation relative to the Co. The value represents *p < 0.05, while **p < 0.005, ***p < 0.001, and ****p < 0.0001 are considered significant, and ns means non-significant.

**FIGURE 10:**
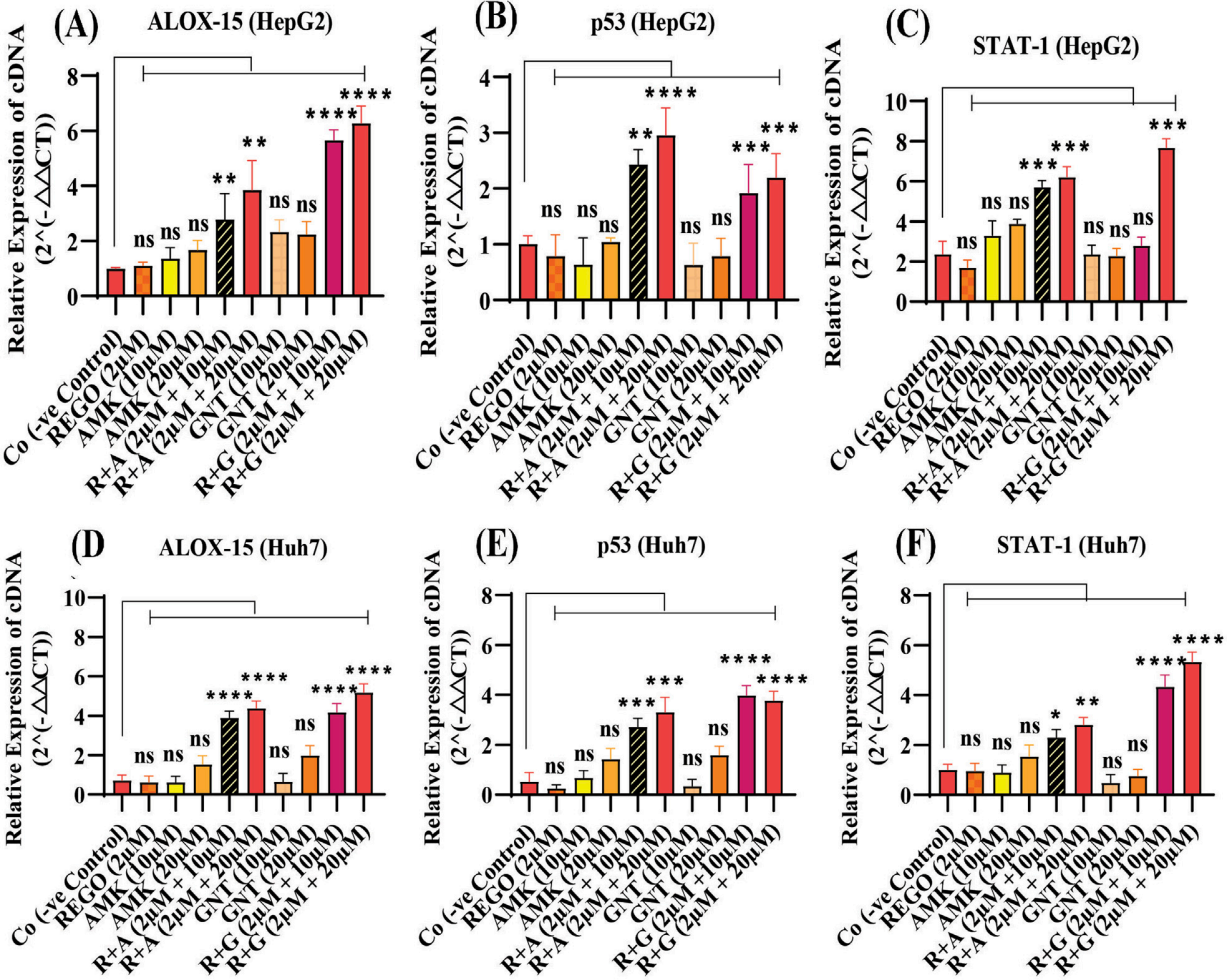
The combination therapy of REGO with AMK and GNT induces ferroptosis by activating ALOX-15, p53, and STAT-1 via an enzymatic pathway. Cells were exposed to respective treatments for 24 h. Subsequently, cDNA was examined using RT-qPCR. **(A)** Fold change of ALOX-15; **(B)** fold change of p53; **(C)** fold change of STAT-1 in HepG2 cells. **(D)** Fold change of ALOX-15; **(E)** fold change of p53; **(F)** fold change of STAT-1 in Huh7 cells. The data are represented as mean ± SD (n = 3). * symbolizes a significant variation relative to the Co. The value represents *p < 0.05, while **p < 0.005, ***p < 0.001, and ****p < 0.0001 are considered significant, and ns means non-significant.

It has been extensively studied that ferroptosis is established through extrinsic and intrinsic pathways. Ample evidence suggests that co-administration of antibiotics with cytotoxic chemotherapeutic drugs exerts their adverse effects, which may cause severe liver injury. Thus, it was hypothesized that REGO and its combination therapy with AMK and GNT might exert the adverse effects associated with ferroptotic cell death. To verify this hypothesis, HepG2 and Huh7 cells were exposed to individual therapies of REGO, AMK, and GNT, as well as in combination with each other at different concentrations. Subsequently, the protein expression in these cells was analyzed through an immunoblotting assay. The results depict that ALOX-15 and associated genes were significantly upregulated in HepG2 and Huh7 cells in the R + A and R + G groups of cells compared to the Co group. In contrast, the cells exposed to individual drug therapy, i.e., REGO, AMK, and GNT, have no significant changes in the protein expression of ALOX-15 (Figures 9A,B). Meanwhile, the genetic expressions of these cells were also examined through RT-qPCR after exposure to respective drugs, and quantification of the fold change of ALOX-15, p53, and STAT-1 genes significantly increased in R + A and R + G groups of cells against negative control. On the other hand, there was no significant mutation in the AMK, REGO, and GNT groups of cells when exposed to the individual therapy of drugs (Figures 10A–F).

## Discussion

The liver is one of the essential organs in the body and is the center of hormonal control, immunological response, nutrition metabolism, and biotransformation of medicinal substances (Trefts et al., 2017). However, the liver performs key functions but is prone to injury (Rad et al., 2024; Velez et al., 2024). It can be damaged due to infection, metabolic disorders, immunity disorders, or drug-induced toxicity (Hoeve et al., 2018). Liver fibrosis, fatty liver (alcoholic or non-alcoholic), and trauma are the leading causes of liver damage. However, acute liver injury is frequently caused by viral infection, ischemia–reperfusion injury, and chemical or drug substances. Chemical or drug-induced liver injury is the term used to describe the damages caused to the hepatocytes or biliary system by the application of xenobiotic chemicals or other pharmacological entities (Senior, 2007). Drug-induced acute liver injury is a frequent and severe clinical illness with high incidence and mortality rates.

Concerning organ damage associated with cell death, recent evidence suggests that ferroptosis is a novel type of regulated cell death. It differs from other types of regulated cell death in terms of the cell structure, metabolism, genetic mutation, and protein expression. Ferroptosis involves three key metabolic processes: thiol, lipid, and iron. These metabolic processes ultimately result in lipid peroxidation, which is dependent on the accumulation of ionic iron inside the cell and ultimately leads to cell death. Ferroptosis has a destructive role and is implicated in the pathologies related to organ damage and degenerative illnesses (Tang et al., 2021; Chen et al., 2022).

In this study, we describe our efforts at using the AG antibiotics, specifically AMK and GNT, which are involved in liver injury when concomitantly administered with REGO, even on clinical therapeutic concentrations. Clinically, AGs are considered the main pharmacological agents used for prophylaxis in various pathophysiological conditions, including post-surgery and cancer-associated immunosuppression (Taplitz et al., 2018; Jayanth et al., 2021). The proposed hypothesis reveals a link between drug toxicity and ferroptosis. Initially, adverse effects of REGO with AMK and GNT were investigated on SD rats. In the initial stage, using blood and liver tissues of SD rats, it was determined that the levels of liver biochemical determinants were increased. Additionally, infiltration and microvascular injury in hepatic tissues were observed following the combination therapy of corresponding drugs (Figure 1). The liver tissues of SD rats were analyzed through RNA-seq; the results revealed that among various DEGs, *Alox-15* was commonly and significantly overexpressed in all groups and associated with the ferroptotic pathway (Figure 3).

Ferroptosis predominantly relies on iron and is marked by elevated levels of LP (Rodriguez et al., 2022). Notably, intracellular Fe^3+^ is reduced to Fe^2+^ by the prostate 6-transmembrane epithelial antigen-3, subsequently integrated into the iron pool, and stored as ferritin (He et al., 2020; Correnti et al., 2024). The Fe^2+^, which is unstable and highly reactive, facilitates the generation of hydroxyl radicals via the Fenton reaction, which can directly interact with polyunsaturated fatty acids (PUFAs) in the cell membrane, resulting in significant elevation in ROS and initiating ferroptosis (Gammella et al., 2016; Chen et al., 2020). ROS encompasses lipid peroxyl radicals, lipid peroxides, and lipid alkoxyl radicals, mainly generated via enzymatic LP or non-enzymatic autoxidation pathways, with the Fenton reaction, which is iron-dependent. Our study determined that MDA levels were elevated in liver tissues of SD rats during Fe^2+^ overload; furthermore, GSH, SOD, and CAT levels were reduced after concomitant use of REGO with AMK and GNT (Figure 2).

However, no single strategy has yet been developed to identify ferroptotic cell death, although different studies have determined multiple techniques for detecting the ferroptosis process (Chen Z., et al., 2024). To demonstrate the presence of ferroptosis in the disease discovery process, cell viability testing is crucial. Overall, research on ferroptosis frequently uses cell status and activity detection through a CCK-8 assay, which is the most popular approach nowadays (Su et al., 2022; Qin et al., 2023; Ma et al., 2024). Moreover, increased LDH release and decreased MMP are also considered to determine ferroptosis (Kumar et al., 2018; Huang et al., 2022; Xu et al., 2022). To discover the further mechanism of liver injury associated with ferroptosis, an *in vitro* assay model was established on HepG2 and Huh-7 cells. Through this, it was determined that cell viability and MMP were decreased in HepG2 and Huh7 cells when these were exposed to the combination therapy of REGO with AMK and GNT compared to individual therapy. Additionally, increased LDH release was observed (Figure 5).

Meanwhile, ROS determination, whether lipid ROS, intracellular ROS, or mitochondrial ROS, is also useful in identifying ferroptosis. Intracellular ROS are frequently detected using DCFH-DA; after staining, green fluorescence is visible with an increase in ROS (Eruslanov and Kusmartsev, 2010). Increased LP is a hallmark of ferroptosis (Dai et al., 2023), which is involved in the oxidative breakdown of PUFA (Tsikas, 2017; Hirschhorn and Stockwell et al., 2019; Wu et al., 2022). While ferroptosis cannot be identified only by elevated ROS and LP, detecting iron levels is also a phenotypic hallmark in the ferroptosis process. Furthermore, GPX4 is an essential regulatory component in the cellular antioxidant defense mechanism (Yang et al., 2014). The key factors regulating ferroptosis are cysteine availability, GSH production, and GPX4 activity (Yang et al., 2016). In the system SLC7A11/XC^−^, glutamate and extracellular cysteine are exchanged in an equal ratio; glutamate is transported outside the cell by solute carrier family 3 member 2, while cysteine is transported intracellularly for GSH production by SLC7A11 (Mandal et al., 2010; Kobayashi et al., 2015). Glutamate, cysteine, and glycine are then converted into GSH by glutamyl cysteine synthetase and glutathione synthetase; as a result, ferroptosis is less likely to occur (Jiang et al., 2021). GSH can enhance the GPX4's interaction with intracellular phospholipid hydroperoxides which will decrease the corresponding phospholipid alcohol levels. Following these parameters, our findings are evident that the combination therapy of AMK and GNT with REGO induces Fe^2+^, MDA, and ROS levels, and GSH production was decreased (Figure 6). Apart from this, the most commonly used methods to validate the occurrence of ferroptosis are RT-qPCR and Western blotting, which confirm the reduced expressions of GPX4 or SLC7A11 (Zhang et al., 2023). The current study proved that the combination therapy of REGO with AMK and GNT decreases the production of GSH, which may downregulate the expressions of GPX4 and SLC7A11, as depicted in Figures 7, 8.

Multiple pathways might be involved in the ferroptosis process; it is still under consideration. However, the two major pathways have been identified: the transporter-dependent pathway or the extrinsic pathway and the intrinsic pathway (Tang et al., 2021). Interestingly, it can be said that both are based on the levels of LP and iron content, which play a key role in the homeostasis of ferroptosis (Rochette et al., 2022). The intrinsic pathway of ferroptosis is regarded as an enzyme-regulated pathway, in which p53 plays a fundamental role in inducing ferroptosis through activation of STAT-1 and ALOX-15 (Jiang et al., 2015). It increases LP production by increasing ROS levels (Ou et al., 2016). Additionally, it can efficiently oxidize diverse phosphatidyl ethanol amines into iron-reducing signaling molecules (Li et al., 2020). PUFAs are highly cationic amines that exhibit strong binding affinity to nucleic acids and are involved in several bimolecular processes, such as DNA replication, translation, and transcription, and these interactions can control the intercellular communication (Casero and Marton, 2007; Han et al., 2024). The enzymatic pathway entails the lipoxygenase enzyme using PUFAs as substrates to generate lipid peroxides, which may damage cellular and organelle membranes (Kuhn et al., 2015; Zheng and Cornad, 2020). The correlation between p53-mediated STAT-1 and ALOX-15 expression has also been observed in cancer cells (Ou et al., 2016). Our investigation revealed that ALOX-15 induces ferroptosis following liver injury, where STAT-1 plays a pivotal role by modulating the LP process and facilitating the elevated production of ALOX-15 in hepatocytes. This study explored the fact that the combination therapy of REGO with AGs induces ferroptosis via activation of ALOX-15 (Figures 9, 10). This indicates that targeting Alox-15 may represent an innovative therapeutic approach for alleviating subsequent damage caused by drug-induced hepatic ferroptosis. Before conducting this study, the clinical significance of the p53/ALOX-15 ferroptotic pathway in sinonasal inverted papilloma was determined (Li et al., 2024). Another study revealed that this pathway plays a vital role in ferroptosis following skeletal muscle contusion (Yang et al., 2024). “Yuan” suggested that this ferroptotic pathway is involved in Alzheimer’s disease (Ren et al., 2024); however, in our novel study, it was explored that activation of ALOX-15 through p53/STAT-1 causes ferroptosis, leading to severe liver damage.

**FIGURE 11:**
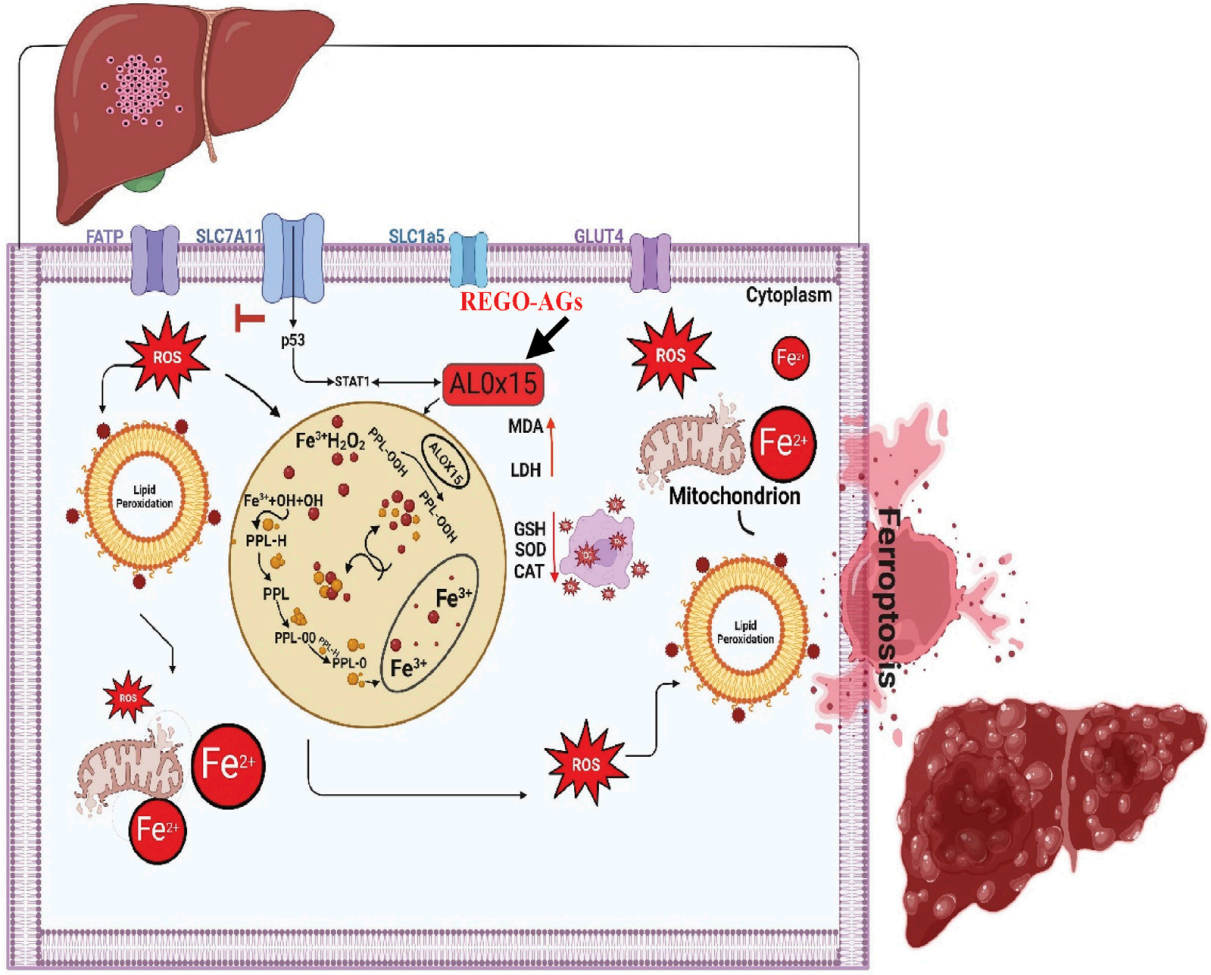
The schematic view of the current study: REGO-AGs activate ALOX-15 (black arrow), which accelerates the Fenton reaction and formation of reactive oxygen species (ROS), lipid peroxidation (LP), and ionic iron (Fe^2+^); ultimately, lactate dehydrogenase (LDH) is released, and malondialdehyde (MDA) level was increased. Antioxidant levels, i.e., glutathione (GSH), superoxide dismutase (SOD), and catalase (CAT), were decreased. Additionally, mitochondrial membrane potential was lost. Finally, cell membrane permeability decreases, and ferroptosis-associated cell death is established. (OH, hydroxyl alcohol; PPL, phospholipid; PPL-OOH, phospholipid hydroperoxide; REGO-AGs, regorafenib with aminoglycosides).

From the above findings, it has been concluded that combination therapy of REGO with AMK and GNT causes significant elevation of Fe^2+^, LP, ROS levels and alteration in protein’s expression, which mediates ferroptotic cell death and leads to liver dysfunction (Figure 11). Specifically, it was first investigated that concomitant use of these drugs activates ALOX-15 through STAT-1, followed by p53, leading to ferroptosis and liver injury.

## Data Availability

The original contributions presented in the study are included in the article/Supplementary Material, further inquiries can be directed to the corresponding author.

## Abbreviations

Alox-15, arachidonic acid 15-lipoxygenase; REGO, regorafenib; AMK, amikacin; GNT, gentamicin; R + A, regorafenib with amikacin; R + G, regorafenib with gentamicin; CMC, carboxymethyl cellulose; ALT, alanine aminotransferase; AST, aspartate aminotransferase; MDA, malondialdehyde; GSH, reduced glutathione; SOD, superoxide dismutase; CAT, catalase; LP, lipid peroxidation; PUFA, polyunsaturated fatty acid; RCD, regulated cell death; DCFH-DA, 2-7 dichlorodihydrofluorescein diacetate; ROS, reactive oxygen species; LDH, lactate dehydrogenase, CCK8, cell count kit 8; SD, Sprague–Dawley; MMP, mitochondrial membrane potential; DMEM, Dulbecco’s modified Eagle medium; FBS, fetal bovine serum.
