# Supplementary material for: Synergistic effect of regorafenib with aminoglycosides in ferroptosis-mediated liver injury
Source: Front Pharmacol. 2025 Jul 15;16:1586578. doi: 10.3389/fphar.2025.1586578 (PMC12303813; doi:10.3389/fphar.2025.1586578)
Supplement: Supplementary file 1 [file DataSheet1.docx]

# Supplementary Material

Supplementary Table S1.1 Antibodies Used for Immunoblotting Assay

| **Category** | **Antibody’s name** | **Company (Cat number)** | **Dilution ratio** |
| --- | --- | --- | --- |
| Primary Antibodies | anti-SLC7A11 | Proteintech (26864-1-AP) | 1:1000 |
|  | anti-GPX4 | Proteintech (67763-1-lg) | 1:1000 |
|  | anti-p53 | Proteintech (60283-2lg) | 1:5000 |
|  | anti-STAT1 | Proteintech (10708-1-AP) | 1:5000 |
|  | anti-ALOX15 | Santa Cruz biotech.(SC133085) | 1:1000 |
|  | Anti-GAPDH | Proteintech (60004-1-IG) | 1:20000 |
| Secondary Antibodies | Anti-Rabbit | EarthOx, UK (E032720-01) | 1:10000 |
|  | Anti-mouse | EarthOx, UK (E032810-01 | 1:10000 |

## Quantitative Real-Time Poly-Chain Reaction (qRT- PCR)

The qRT-PCR reaction system was prepared according to components and volume specified in the Supplementary Table. S1.2. For amplification of qRT-PCR, the reaction procedure was fixed as: Initial Denaturation @ 95 degrees Celsius for five minutes **>** Further Denaturation @ 90 degrees Celsius for thirty seconds > Annealing @ 58 degrees Celsius for only 30 seconds > Extension at 72 degrees Celsius for 30 seconds > Ending Termination at 4 degrees Celsius. Note: the amplification process was repeated for a total of 38 cycles.

Supplementary Table S1.2 Components of qRT-PCR protocol

| Components | Volume Used |
| --- | --- |
| cDNA | 1-μl |
| Primer (F&R) | 0.5 × 2μl |
| Double Distilled water | 3-μl |
| SYBR Green Mixture | 5-μl |
| Total | 10 μl (For one replicate) |

Note this concentration was multiplied by number of replicates used accordingly.

Table S1.3A Primers Used for RT-qPCR

| **Name Of Primers** | **Forward Sequencing Of Primers Used** | **Reverse Sequencing Of Primers Used** |
| --- | --- | --- |
| rAlox15 | CATCGGAGACTCCAAGTACG | GAGTTCTGCTTCCGAGTCAA |
| rIgfbp1 | TTTATCACAGCAAACAGTGCGA | CTTCCCACTCCATGGGTAGAC |
| rTrim36 | AAAGGGTCTTGACCATGTCGG | TTCTTGATAGTCACCTTGCCCT |
| rGAPDH | CCGCCATCTTCTTGTGCAGTG | CCGATACGGCCAAATCCGTT |

Note: r represents the genus Rattus.

Supplementary Table S1.3B Primers and Their Sequences Used for RT- qPCR

| **Name Of Primers** | **Forward Sequencing Of Primers** | **Reverse Sequencing Of Primers** |
| --- | --- | --- |
| hAlox15 | CACCGGAGACTCCAAGTACG | TGAATTCTGCTTCCGAGTCC |
| hSlc7A11 | TGGAACTTCACCAAGTTTGGA | GGGCAGGTCCTTCTCTATCA |
| hGPX4 | AGCGCTATAGTGTTCACAGGT | AGGTGGAGAATTGAGAGCACG |
| hSTAT-1 | GCTCGTTTGTGGTGGAAAGAC | TCTCTCATTCACATCTCTCAACTT |
| hp53 | AGTCTAGAGCCACCGTCCAG | AAGGCAGTCTGGCCAATCC |
| hGAPDH | AAGCCTGCCGGTGACTAAC | CGCCCAATACGACCAAATCAGA |

Note: h represents homo sapien
